# Comorbidities associated with nontuberculous mycobacterial disease in Japanese adults: a claims-data analysis

**DOI:** 10.1186/s12890-020-01304-6

**Published:** 2020-10-09

**Authors:** Shunsuke Uno, Takanori Asakura, Kozo Morimoto, Kimio Yoshimura, Yoshifumi Uwamino, Tomoyasu Nishimura, Yoshihiko Hoshino, Naoki Hasegawa, Nontuberculous Mycobacteriosis and Bronchiectasis-Japan Research Consortium (NTM-JRC)

**Affiliations:** 1 Department of Infectious Diseases, Keio University School of Medicine, 35 Shinanomachi, Shinjuku, Tokyo, Japan; 2 Division of Pulmonary Medicine, Department of Medicine, Keio University School of Medicine, Tokyo, Japan; 3 Department of Mycobacteriology, Leprosy Research Center, National Institute of Infectious Diseases, Tokyo, Japan; 4 The Research Institute of Tuberculosis, Japan Anti-Tuberculosis Association, Tokyo, Japan; 5 Division of Clinical Research, Fukujuji Hospital, Japan Anti-Tuberculosis Association, Tokyo, Japan; 6 Department of Health Policy and Management, Keio University School of Medicine, Tokyo, Japan; 7 Department of Laboratory medicine, Keio University School of Medicine, Tokyo, Japan; 8 Keio University Health Center, Tokyo, Japan

**Keywords:** Claims-data analysis, Comorbidities, Nontuberculous mycobacterial diseases

## Abstract

**Background:**

Nontuberculous mycobacterial (NTM) lung disease is one of a growing number of chronic health problems that is difficult to cure in aging societies. While it is important to be vigilant about associated comorbidities in order to provide better patient care, data on the prevalence of comorbidities stratified by country or region are scarce. We aimed to elucidate the comorbidities associated with NTM disease based on Japanese health insurance claims data.

**Methods:**

Cross-sectional analyses were performed using the claims data for 2014 provided by the Japan Medical Data Center Co., Ltd. Patients aged 20–75 years with ≥3 claims associated with NTM disease were identified and matched to 10 sex-and-age-matched controls that had never made a claim for NTM disease. Thirty-one comorbidities previously suspected to be associated with NTM disease were selected, and the prevalence of these comorbidities compared between cases and controls.

**Result:**

Overall, 419 NTM patients (134 males and 285 females) and 4190 non-NTM controls were identified from the JMDC database. Aspergillosis, asthma, chronic heart failure, diffuse panbronchiolitis, gastroesophageal reflux, interstitial pneumonia, lung cancer, cancer other than breast, lung, ovary, or prostate cancer, and rheumatoid arthritis were associated with NTM disease in both males and females. Chronic obstructive pulmonary disease was associated with NTM in males while chronic kidney disease, osteoporosis, and Sjögren syndrome were associated with NTM in females.

**Conclusion:**

NTM disease was associated with multiple comorbidities that should be considered when providing medical care to individuals with NTM disease.

## Background

Nontuberculous mycobacterial (NTM) lung disease is one of a growing number of chronic health problems that is difficult to cure. Some patients remain stable without treatment, but others die despite being treated with multidrug combination antimycobacterial chemotherapy. The incidence of NTM lung disease is rapidly increasing worldwide [1], and lifelong antimycobacterial chemotherapy is often required to control the disease.

Patients with chronic NTM disease, regardless of whether they are on treatment or not, are getting older and their quality of life may be lowered [2]. In the Japanese aging society, patients with NTM disease have become older, and prevalence of the NTM disease is higher in older people. Individuals with NTM disease often suffer from other health problems such as malignancies or cardiovascular diseases, and these comorbidities are often the cause of death [3]. It is important to pay attention to comorbidities in the management of NTM disease in order to provide optimal patient care.

Previous retrospective studies have shown that diffuse panbronchiolitis, lung cancer, and rheumatoid arthritis may be associated with NTM disease [4–6]. Population-based studies have also identified comorbidities such as chronic obstructive pulmonary diseases (COPD), osteoporosis, gastroesophageal reflux (GERD), cystic fibrosis to be associated with NTM disease [7, 8]. Marras et al. [9] analyzed the comorbidities associated with NTM disease using claims data of the United States (US) managed-care population, which included both NTM and non-NTM patients. However, disease prevalence should be analyzed on the basis of the data of each country or region because differences in environmental and genetic factors lead to different comorbidities (e.g., the prevalence of atherosclerosis or cardiovascular disease differs in each country or region) [10, 11]. In addition, the pathogenic species of *Mycobacterium* vary by country and region [8, 12] and the prevalence of different species may lead to the different clinical picture. Therefore, we conducted claims-data-based analyses to determine the comorbidities associated with NTM disease in Japan.

## Methods

### Data source

Analyses were conducted using claims data provided by the Japan Medical Data Center Co., Ltd. (JMDC). JMDC has contracts with multiple Japanese health insurance societies and has accumulated reimbursement data from more than 3,000,000 individuals. The JMDC database includes only individuals aged < 75 years because individuals aged ≥75 years are covered by National Health Insurance and membership of the original health insurance society is terminated on reaching the age of 75 years. Personal information is encrypted and claims data are recorded chronologically for each individual under a unique encrypted identifier. The database records information on all medical consultations from all medical institutions and medical care providers. The information about residence of the enrollees are deleted in the database. Seven hundred sixty-nine thousand six hundred twenty-seven individuals who were registered in JMDC in 2014 were employees and 794,669 were dependent family members. Health insurance societies that had contracts with JMDC did not include business owners or welfare recipients.

### Study design, patient identification, and matching

A case-control study was conducted using JMDC data. Individuals with NTM disease were included from 1,564,296 individuals who were registered in JMDC in 2014. Individuals aged ≥20 years with ≥3 claims associated with NTM disease (International Classification of Diseases, tenth revision [ICD-10] A31.0 [Pulmonary mycobacterial infection] or A31.9 [Msycobacterial infection, unspecified]) on separate occasions ≥1 month apart from January 2014 to December 2014 were identified from the JMDC data. The prevalence of NTM disease in 2014 was calculated. Controls that never had a claim associated with NTM disease were randomly extracted from patients who visited a medical facility at least once in 2014. A total of ten controls were matched by sex and age for each case.

Other comorbidities were identified utilizing ICD-10 codes from medical claims based on ≥3 claims separate occasions ≥1 month apart in 2014 as well (Table 1). Only confirmed diagnoses were included in the analysis. The 30 comorbidity complexes that had previously been suspected as associated with NTM disease were selected and compared [4–9, 13, 14]. The definition of diffuse panbronchiolitis (DPB) also required be on long-term macrolide treatment.

**Table 1:** ICD-10 codes for definition of comorbidity complexes

Comorbidity complexes	ICD-10 codes
Arrhythmia	I44.x, I45.x, I47.x, I48.x, I49.x
Aspergillosis	B44.x
Asthma	J45.x
Bone fracture	M80.x, M84.0, M84.3, M84.4, M96.6, S02.x, S22.x, S32.x, S42.x, S52.x, S62.x, S72.x, S82.x, S92.x, T02.x, T08, T14.2, T91.1, T93.2, T94.1
Breast cancer	C50.x
Chronic heart failure	I11.x, I42.x, I50.x
Chronic kidney disease	N18.x, N28.9
COPD	J43.x, J44.9
Crohn’s disease	K50.x
Depression	F32.x, F33.x
Diabetes mellitus	E10.x-E14.x, R73.0
Diffuse panbronchiolitis	J44.8
Dyslipidemia	E78.x
GERD	K21.0, K21.9
Heart valve disease	I05.x-I08.x, I34.x-I37.x, I38
HIV infection	B24
Hypertension	I10, I11.x, I12.x, I15.x
Ischemic heart disease	I20.x, I21.x, I24.x, I25.1, I25.2, I25.5, I25.6, I25.9
Interstitial pneumonia	J84.1, J84.9, J70.4
Liver cirrhosis	B18.1, B18.2, K70.3, K74.6
Lung cancer	C34.x
Osteoporosis	M80.x, M81.x
Other cancer	C00.x-C26.x, C30.x-C33.x, C37.x-C41.x, C43.x-C49.x, C51.x-C55.x, C57.x, C58.x, C60.x, C62.x-C85.x, C88.x, C90.x-C97.x
Ovary cancer	C56
PM/DM	M33.x
Prostate cancer	C61
Rheumatoid arthritis	M06.9
Sjogren syndrome	M35.0
SLE	M32.1, M32.9
Systemic sclerosis	M34.x

*COPD* Chronic obstructive pulmonary diseases, *GERD* Gastroesophageal reflux, *HIV* Human immunodeficiency virus, *PM/DM* Polymyositis/dermatomyositis, *SLE* Systemic lupus erythematosus

Ethics approval was not applicable to this study based on Ethical Guidelines for Medical and Health Research Involving Human Subject issued by the Japanese Ministry of Health, Labour and Welfare since only completely encrypted data were used.

### Statistical analysis

The proportion of comorbidities among NTM group and non-NTM group was compared by chi-square test. We addressed multiple testing by setting a Bonferroni-adjusted significance level of *P*-value < 0.000833 (0.05/60) as we tested 30 comorbidity complexes separating sex. All statistical analyses and matching were performed using EZR (Saitama Medical Center, Jichi Medical University, Saitama, Japan), graphical user interface for R (The R Foundation for Statistical Computing, Vienna, Austria).

## Results

**Fig. 1:**
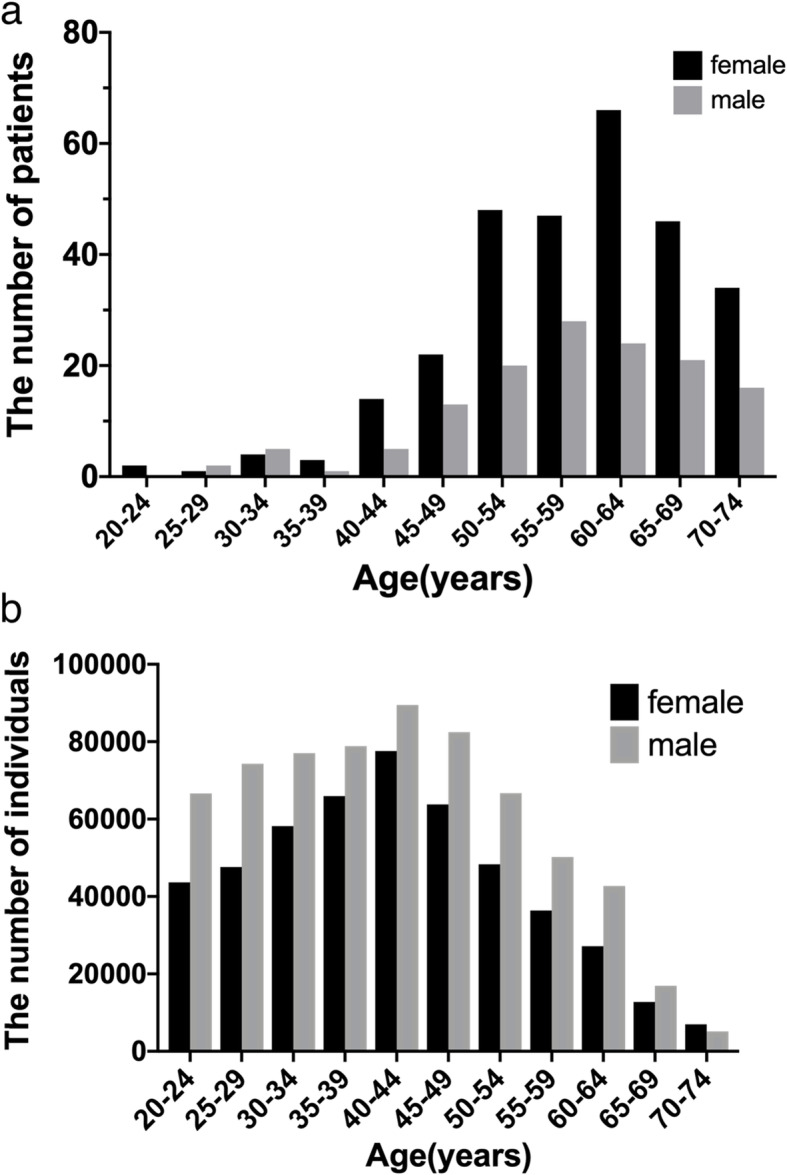
**a** The distribution of the patients with NTM disease by sex and age. **b** The distribution of entire individuals who were registered in JMDC’s database in 2014

A total of 419 NTM patients (134 males and 285 females) were selected from the JMDC database, and 4190 non-NTM controls (1340 males and 2850 females) were identified. The sex and age distribution of the patients is shown in Fig. 1. The median age was 59 years (males 58, females 60); 68% (285/419) were female. Overall, 1,564,296 people were registered in JMDC in the year 2014, and the prevalence of NTM diseases in the patients aged 20–75 years old was calculated as 26.8 /100,000 population.

The prevalence of comorbidities is shown in Tables 2 and 3. Of the respiratory diseases considered, aspergillosis, asthma, DPB, interstitial pneumonia, and lung cancer were significantly associated with NTM disease in both sex groups. In addition, GERD, other cancer, and rheumatoid arthritis were significantly associated with NTM disease in both sex groups. COPD was also significantly associated with NTM disease in males, while chronic kidney disease (CKD), osteoporosis, and Sjögren syndrome were significantly associated with NTM disease in females. The prevalence of chronic heart failure was significantly associated with NTM disease in the unstratified analysis, but not in the sex-stratified analysis. For patients with DPB, six were on long-term macrolide treatment and one was treated with multidrug anti-NTM antibiotics including macrolides.

**Table 2:** NTM disease and Comorbidity complexes

Comorbidity complexes	NTM(*N* = 419) n (%)	non-NTM (*N* = 4190) n (%)	Odds ratio [95% CI]	*P* value
Arrhythmia	28 (6.68)	176 (4.20)	1.63 [1.04–2.48]	0.0243
Aspergillosis	8 (1.90)	0 (0)	+∞ [17.3 − +∞]	< 0.0001*
Asthma	77 (18.3)	194 (4.63)	4.63 [3.43–6.21]	< 0.0001*
Bone fracture	13 (3.10)	64 (1.52)	2.06 [1.03–3.83]	0.0255
Breast cancer	4 (0.954)	43 (1.02)	0.93 [0.24–2.58]	> 0.99
Chronic heart failure	31 (7.39)	130 (3.10)	2.49 [1.61–3.77]	< 0.0001*
Chronic kidney disease	16 (3.81)	54 (1.28)	3.04 [1.61–5.45]	0.000403*
COPD	13 (3.10)	22 (0.525)	6.06 [2.78–12.69]	< 0.0001*
Crohn’s disease	2 (0.477)	0 (0)	+∞ [1.88 − +∞]	0.00825
Depression	16 (3.81)	107 (2.55)	1.51 [0.83–2.61]	0.149
Diabetes mellitus	62 (14.7)	608 (14.5)	1.02 [0.76–1.36]	0.884
Diffuse panbronchiolitis	7 (1.67)	0 (0)	+∞ [14.57 − +∞]	< 0.0001*
Dyslipidemia	105 (25.0)	1110 (26.4)	0.93 [0.73–1.17]	0.561
GERD	83 (19.8)	355 (8.47)	2.67 [2.02–3.49]	< 0.0001*
Heart valve disease	11 (2.62)	54 (1.28)	2.06 [0.97–4.03]	0.0455
HIV infection	0 (0)	0 (0)	NA	NA
Hypertension	110 (26.2)	1166 (27.8)	0.92 [0.73–1.16]	0.529
Ischemic heart disease	29 (6.92)	191 (4.55)	1.56 [1.00–2.35]	0.04
Interstitial pneumonia	25 (5.96)	14 (0.334)	18.89 [9.36–39.70]	< 0.0001*
Liver cirrhosis	4 (0.954)	25 (0.596)	1.61 [0.40–4.68]	0.33
Lung cancer	19 (4.53)	13 (0.310)	15.24 [7.08–33.86]	< 0.0001*
Osteoporosis	46 (10.9)	220 (5.25)	2.23 [1.56–3.13]	< 0.0001*
Other cancer	41 (9.78)	117 (2.79)	3.77 [2.54–5.52]	< 0.0001*
Ovary cancer	1 (0.238)	8 (0.190)	1.25 [0.028–9.37]	0.576
PM/DM	1 (0.238)	2 (0.0477)	5.01 [0.085–96.47]	0.249
Prostate cancer	0 (0)	15 (0.357)	0 [0–2.79]	0.389
Rheumatoid arthritis	23 (5.48)	66 (1.57)	3.63 [2.13–5.99]	< 0.0001*
Sjögren syndrome	10 (2.38)	13 (0.310)	7.85 [3.06–19.50]	< 0.0001*
SLE	3 (0.715)	7 (0.167)	4.31 [0.72–18.95]	0.0552
Systemic sclerosis	4 (0.954)	3 (0.0715)	13.44 [2.26–91.95]	0.00189

*COPD* Chronic obstructive pulmonary diseases, *GERD* Gastroesophageal reflux, *HIV* Human immunodeficiency virus, *PM/DM* Polymyositis/dermatomyositis, *SLE* Systemic lupus erythematosus, *statistically different, *NA* Not applicable

**Table 3:** NTM disease and Comorbidity complexes analyzed separating sexes

	Male *N* = 1474				Female *N* = 3135			
Comorbidity complexes	NTM, *N* = 134	non-NTM, *N* = 1340	Odds ratio [95% CI]	P value	NTM, *N* = 285	non-NTM, *N* = 2850	Odds ratio [95% CI]	P value
Arrhythmia	15 (11.19)	71 (5.29)	2.25 [1.16–4.12]	0.0105	13 (4.56)	105 (3.68)	1.25 [0.64–2.27]	0.417
Aspergillosis	5 (3.73)	0 (0)	+∞ [9.36 − +∞]	< 0.0001*	3 (1.05)	0 (0)	+∞ [4.15 − +∞]	0.000744*
Asthma	30 (22.3)	60 (4.47)	6.14 [3.65–10.17]	< 0.0001*	47 (16.4)	134 (4.70)	4.00 [2.73–5.78]	< 0.0001*
Bone fracture	3 (2.23)	8 (0.597)	3.81 [0.64–16.11]	0.0705	10 (3.50)	56 (1.96)	1.81 [0.82–3.64]	0.0854
Breast cancer	0 (0)	1 (0.0746)	0 [0–387.6]	> 0.99	4 (1.40)	42 (1.47)	0.95 [0.25–2.65]	> 0.99
Chronic heart failure	11 (8.20)	43 (3.20)	2.69 [1.22–5.49]	0.00731	20 (7.01)	87 (3.05)	2.40 [1.37–4.01]	0.00158
Chronic kidney disease	5 (3.73)	25 (1.86)	2.04 [0.60–5.54]	0.185	11 (3.85)	29 (1.01)	3.90 [1.74–8.16]	0.000605*
COPD	9 (6.71)	11 (0.820)	8.67 [3.11–23.51]	< 0.0001*	4 (1.40)	11 (0.385)	3.67 [0.85–12.49]	0.0408
Crohn’s disease	2 (1.49)	0 (0)	+∞ [1.89 − +∞]	0.0821	0 (0)	0 (0)	NA	NA
Depression	2 (1.49)	32 (2.38)	0.62 [0.071–2.48]	0.763	14 (4.91)	75 (2.63)	1.91 [0.98–3.47]	0.0374
Diabetes mellitus	28 (20.89)	241 (17.98)	1.20 [0.75–1.89]	0.412	34 (11.9)	367 (12.8)	0.92 [0.61–1.34]	0.71
Diffuse panbronchiolitis	4 (2.98)	0 (0)	+∞ [6.70 − +∞]	< 0.0001*	3 (1.05)	0 (0)	+∞ [4.15 − +∞]	0.000744*
Dyslipidemia	26 (19.4)	345 (25.7)	0.69 [0.43–1.10]	0.118	79 (27.7)	765 (26.8)	1.05 [0.79–1.38]	0.779
GERD	35 (26.1)	118 (8.80)	3.66 [2.31–5.71]	< 0.0001*	48 (16.8)	237 (8.31)	2.23 [1.56–3.15]	< 0.0001*
Heart valve disease	6 (4.47)	17 (1.26)	3.64 [1.16–9.90]	0.0139	5 (1.75)	37 (1.29)	1.36 [0.41–3.50]	0.427
HIV infection	0 (0)	0 (0)	NA	NA	0 (0)	0 (0)	NA	NA
Hypertension	40 (29.8)	432 (32.2)	0.89 [0.59–1.33]	0.628	70 (24.5)	734 (25.7)	0.94 [0.70–1.25]	0.722
Ischemic heart disease	14 (10.4)	75 (5.59)	1.97 [1.00–3.64]	0.0344	15 (5.26)	116 (4.07)	1.31 [0.70–2.29]	0.35
Interstitial pneumonia	12 (8.95)	4 (0.298)	32.66 [9.71–141.0]	< 0.0001*	13 (4.56)	10 (0.350)	13.55 [5.43–34.89]	< 0.0001*
Liver cirrhosis	3 (2.23)	3 (0.223)	10.17 [1.35–76.71]	0.012	1 (0.350)	22 (0.771)	0.45 [0.011–2.82]	0.716
Lung cancer	10 (7.46)	8 (0.597)	13.37 [4.66–39.77]	< 0.0001*	9 (3.15)	5 (0.175)	18.52 [5.53–70.69]	< 0.0001*
Osteoporosis	4 (2.98)	10 (7.46)	4.09 [0.92–14.42]	0.0318	42 (14.7)	210 (7.36)	2.17 [1.48–3.13]	< 0.0001*
Other cancer	18 (13.4)	45 (3.35)	4.46 [2.35–8.16]	< 0.0001*	23 (8.07)	72 (2.52)	3.39 [1.98–5.59]	< 0.0001*
Ovary cancer	0 (0)	0 (0)	NA	NA	1 (0.35)	8 (0.280)	1.25 [0.028–9.39]	0.576
PM/DM	0 (0)	0 (0)	NA	NA	1 (0.35)	2 (0.0701)	5.01 [0.085–96.61]	0.249
Prostate cancer	0 (0)	15 (1.11)	0 [0–2.79]	0.387	0 (0)	0 (0)	NA	NA
Rheumatoid arthritis	6 (4.47)	5 (0.373)	12.47 [3.12–52.32]	0.00016*	17 (5.96)	61 (2.14)	2.90 [1.56–5.12]	0.000458*
Sjögren syndrome	0 (0)	0 (0)	NA	NA	10 (3.50)	13 (0.456)	7.92 [3.08–19.76]	< 0.0001*
SLE	0 (0)	1 (0.0746)	0 [0–387.6]	> 0.99	3 (1.05)	6 (0.210)	5.04 [0.81–23.74]	0.0413
Systemic sclerosis	1 (0.746)	1 (0.0746)	10.03 [0.13–785.3]	0.174	3 (1.05)	2 (0.0701)	15.11 [1.72–181.5]	0.0647

*COPD* Chronic obstructive pulmonary diseases, *GERD* Gastroesophageal reflux, *HIV* Human immunodeficiency virus, *PM/DM* Polymyositis/dermatomyositis, *SLE* Systemic lupus erythematosus, *statistically different, *NA* Not applicable

## Discussion

We conducted a case-control study using JMDC data and investigated the coexistence of NTM disease and comorbid conditions in Japanese adults. JMDC has collected reimbursement data since 2005 and the size of the database makes it valuable resource for estimating the prevalence of diseases. This is the first study evaluating comorbidities associated with NTM disease in Japanese adults based on a case-control analysis using Japanese health insurance claims database. We have newly added comorbidities such as CKD, DPB, osteoporosis, and Sjögren syndrome to previously reported comorbidities in the US claims-data analysis [9]. Human immunodeficiency virus (HIV) and cystic fibrosis are well-known risk factors of NTM disease [15]. However, our data did not contain individuals with these diseases because of their low prevalence in Japan. The calculated prevalence and the proportion of females with NTM disease were similar to those reported in a previous Japanese study [14] of NTM lung disease (NTM-LD). It is likely that the majority of cases in our study had NTM-LD despite of definition it in two codes, A31.0 and A31.9, which are used essentially interchangeably in Japan. The database in JMDC was not contain the result of culture or the residence of individuals; therefore, the analyses including those information was unavailable in this study. A strength of this study is that it included 10 sex-and-age-matched non-NTM controls per case. In addition, we performed Bonferroni-adjustment to resolve the problem of multiple hypothesis testing.

Respiratory diseases and a variety of systemic conditions are comorbid with NTM disease. In terms of respiratory diseases, Marras et al. [9, 16] found an association between NTM disease and aspergillosis, asthma, COPD, lung cancer, and tuberculosis in the US claims-data analysis. Our data showed that interstitial lung disease and asthma were comorbid with NTM disease in addition to the classical risk factors such as COPD. A Korean study with 810 NTM-LD patients revealed that 42 patients (5.2%) had interstitial lung disease, which is a similar proportion to that found in our study [17]. Asthma and COPD, especially if treated with inhaled corticosteroid therapy, and the use of systemic immunosuppressive agents have previously been identified as strongly associated with NTM-LD [8, 9, 18, 19], which might also contribute to the development of NTM-LD in individuals with interstitial lung disease.

Tsuji et al. [4] conducted a retrospective study of 33 patients with diffuse panbronchiolitis (DPB) and found that 7 (21.2%) of the patients also had pulmonary NTM disease. In our analysis, 1.9% of patients with NTM disease also had DPB. This fact is more characteristic in Japanese or Asian patients than it is among individuals in the US or Europe because DPB mainly affects East Asian people [20]. We speculated that DPB is a risk factor for NTM disease, but does not occur due to NTM disease. *Mycobacterium* spp. also easily colonize the respiratory tract of DPB patients because DPB is accompanied mucociliary dysfunction and various bacteria colonize the respiratory epithelium [4]. NTM is a well-recognized pathogen in cystic fibrosis patients [21, 22]. However, the prevalence of cystic fibrosis in Japan is very limited, and there were no patients with cystic fibrosis in our data.

Kusumoto et al. [5] pointed out the high incidence of lung cancer in patients with NTM-LD, with an estimated incidence rate of 124.6 per 100,000 patient-years. This incidence is much higher than that reported in Japan. These association between NTM diseases and lung cancer could be a detection bias, as patients with NTM-LD have chest CT scans more frequently than healthy individuals and are therefore more likely to have other lung nodules discovered incidentally. Kusumoto et al. also speculated that chronic lung inflammation might cause lung cancer, however, the pathogenesis of lung cancer in relation to NTM disease is unknown [5].

In terms of non-pulmonary comorbidities, patients with NTM had a significantly higher prevalence of chronic heart failure, GERD, cancer other than breast, lung, ovary, or prostate cancer, and rheumatoid arthritis than controls in both males and females. Females with NTM disease also had a significantly higher prevalence of CKD, osteoporosis, and Sjögren syndrome than controls. The coexistence between NTM disease and chronic heart failure, GERD, other cancer, and rheumatoid arthritis has been reported previously [8, 9, 16]. Chao et al. [23] reported an increased risk of NTM disease in patients with Sjögren syndrome based on an analysis of Taiwan’s National Health Insurance Research Database analysis. Pulmonary abnormalities have been described as an extra-glandular involvement of Sjögren syndrome [24]. NTM-LD may be an underlying condition in patients with Sjögren syndrome because some patients with Sjögren syndrome have been reported to develop bronchiectasis [25, 26], and pulmonary involvement in patients with Sjögren syndrome can be difficult to distinguish from NTM-LD using CT.

Menopause is a well-known risk factor for NTM disease [27], and osteoporosis also frequently occur in those population. Low serum estradiol had been related to development of NTM-LD [28], and it might be a confounding factor between osteoporosis and NTM diseases. In addition, Jeon et al. [13] reported the association between severe vitamin D deficiency and development of NTM disease; thus, vitamin D deficiency may also be a confounding factor of these diseases. Measurement of serum vitamin D level was not supported by the health insurance system in Japan in 2014, so we were unable to assess whether there was an association between vitamin D deficiency and NTM disease. COPD, a common chronic pulmonary disease, has been reported to be an independent risk factor of osteoporosis [29] and so there is a need to investigate the mechanism of development of osteoporosis in patients with NTM disease.

This study should be interpreted in light of several limitations. First, the analysis was based on data from a claims database. Since we did not have access to the clinical or microbiological information of the participants, the prevalence of some disease complexes might differ from that based on a true diagnosis and it needs to be interpreted with caution and would be preferred to be validated by clinical study. Previous researchers have defined their targeted diseases by ≥2 claims in several claims-data analyses [9, 16, 30–33]; however, we defined by ≥3 claims to increase the specificity. Our more stringent definition of comorbidities may have led to a bias toward the null, making it less likely to detect true associations. However, this feature of our study likely ensured that the detected associations were accurate and robust. This issue is further strengthened by the use of Bonferroni correction for multiple comparisons. The calculated prevalence and proportion of women were similar to that reported in the previous study [14]; thus, the accuracy of our diagnosis of NTM disease is likely to be comparable to that of previous studies. Second, we were unable to differentiate the *Mycobacterium* species due to unavailability of microbiological information. Third, JMDC database did not include patients aged ≥75 years belonging to National Health Insurance. Thus, the result should be interpreted as people < 75 years of age and the association of older patients could differ. It would be ideal to have included patients aged ≥75 years as comorbidities increases with advanced age. In addition, there is another selection bias of JMDC database including only employee and their dependent family members and not including business owners or welfare recipients. Fourth, it should be interpreted to be careful to the differences with clinical significance in such a big data analysis instead of performance of Bonferroni-adjusted analysis. In addition, the prevalence of aspergillosis or DPB are 0 in non-NTM group; thus, it should be validated with larger datasets.

## Conclusion

we elucidated the comorbidities complex associated with NTM disease in Japanese adults based on a claims-data analysis. Clinicians should be aware of these specific comorbidities when providing medical care for patients with NTM disease.

## Data Availability

The datasets used and analyzed during the current study are available from the corresponding author on reasonable request.

## Abbreviations

CKD: Chronic kidney disease
COPD: Chronic obstructive pulmonary diseases
CT: Computed tomography
DPB: Diffuse panbronchiolitis
GERD: Gastroesophageal reflux
HIV: Human immunodeficiency virus
ICD-10: International Classification of Diseases, tenth revision
JMDC: Japan Medical Data Center Co., Ltd.
NTM: Non-tuberculous *Mycobacterium*
NTM-LD: Non-tuberculous *Mycobacterium* lung disease
PM/DM: Polymyositis/dermatomyositis
SLE: Systemic lupus erythematosus
US: United States
